# Supplementary material for: Sharp increase in inequality in education in times of the COVID-19-pandemic
Source: PLoS One. 2022 Feb 2;17(2):e0261114. doi: 10.1371/journal.pone.0261114 (PMC8809564; doi:10.1371/journal.pone.0261114)
Supplement: Attachment — Submitted filename: Response to reviewers.docx [file pone.0261114.s001.docx]

**Sharp increase in inequality in education in times of the COVID-19-pandemic**

**PLOS ONE**

September 2021

*First, let us express our gratitude for your and the reviewers’ constructive comments and remarks. They have helped us a lot in revising and essentially improving the paper. We believe that we have been able to address all of your comments. We hope that you will be satisfied by the respective revisions and replies. Below is a point-by-point response to the main comments that you stressed in the decision email.*

**Response to main points mentioned by the editor**

Please ensure that your manuscript meets PLOS ONE's style requirements, including those for file naming.

*Thank you for reminding us about this and our apologies that we did not have this before. We have now adjusted the manuscript to the journals style requirements, including the referencing style.*

In the Methods section and the online submission form, please provide additional information about the participant records used in your study. Specifically, please ensure that you have discussed whether all data were fully anonymized before you accessed them.

*We have now added the following to the manuscript on page 7:*

*“Note that the data in the environment of Statistics Netherlands are pseudonymized such that data are fully anonymous to the researchers that use these data. The pseudonymization key is only known to Statistics Netherlands and they provide separate datasets with the same person identifier, such that the data can be matched, but individuals cannot be identified by the researchers.”*

Please remove any funding-related text from the manuscript and let us know how you would like to update your Funding Statement.

*Thank you for pointing out that we did not do this in the correct way. We have now removed the funding related text from the manuscript. The Funding statement already was and still is complete, so that does not need to be changed.*

In your Data Availability statement, you have not specified where the minimal data set underlying the results described in your manuscript can be found. PLOS defines a study's minimal data set as the underlying data used to reach the conclusions drawn in the manuscript and any additional data required to replicate the reported study findings in their entirety.

*Our apologies, it seems that something went wrong with the data availability statement. We have adjusted that now. Please let us know should the current data availability statement not be sufficient.*

Please review your reference list to ensure that it is complete and correct.

*­­­*

*Thank you. We have now updated our reference list, and made sure that it’s complete and correct.*

**Response to reviewer 1**

*First, let us express our gratitude for your positive words, and constructive comments and remarks. They have helped us a lot in revising and essentially improving the paper. We believe that we have been able to address all of your and their comments. We hope that you will be satisfied by the respective revisions and replies.*

In general, the article looks interesting and gives good and useful information.

*Thank you.*

There are, however, some minor points that must be corrected before publication.

One of the most important arguments of this article, compared to previous similar publications is:
"we look in greater detail at background differences between students and present results showing that the learning loss due to the school closures are unequally distributed and that students from disadvantaged backgrounds have suffered much more than their fellow students."
I would like to understand better what is the meaning of "greater detail" since a new publication should offer something different and it should be clearly explained.

*Thank you for this comment. We should indeed have been more specific in discussing our* *contribution to the existing literature with this study. We have now added some additional explanation to the manuscript, and the text on page 3 we have now added the following:*

*“In comparison with the study that is most similar that also uses Dutch data (Engzell et al. 2020), our study complements and improves the findings for several reasons. 1) We have a larger sample (18% of all Dutch primary schools, instead of 15%), 2) we have much better and richer student background information at the individual level (instead of socio-economic status measured at the neighborhood level, which is very imprecise), 3) we have multiple student background variables that indicate whether a student is disadvantaged or not, and 4) we focus on effects for separate grade levels, showing large variation there, instead of only looking at overall effects.**”*

Getting data from 2018 regarding internet access in Dutch households seems to be a source of information that can be updated for sure with more recent data sources.

*Thank you for pointing this out. You are right, and we have now replaced the source by the most recently available data, which is from 2020. The share of households with internet access is the same though, so we did not change the percentage in the text.*

Materials and methods: Standardized tests had the same format in the pandemic? Were they online or face-to-face?

*Yes indeed, the standardized test during the pandemic were exactly the same as before the pandemic. For most schools, these are digital tests that take place at school. Some schools opt for the pen-and-paper version. But there is there is no within school variation between type and format of the tests before and during the pandemic. We have now also added this information to the manuscript on page 6.*

I think authors should (if possible) to describe a little bit better the correction CITO made related to the delay in the tests. This fact is very important because authors are analyzing differences in those tests, actually. We must be sure that there is not any influence in that sense.

*You are raising a fair point here. Unfortunately, the details of the correction that CITO has applied to these tests are only known to CITO, and not shared with schools or researchers using these data. However, CITO have a long history of excellent expertise in test development, calibration and correction, so we trust in CITOs many years of experience in these matters.*

*Having said that, we did include a dummy in all our analyses whether a student participated in a test before or after the summer break, and this does not influence our results. Furthermore, we have also checked whether we find similar results when we only focus on the students that wrote the test before the summer break (so in the regular time frame of the test), and we do not find different results or draw different conclusions. Therefore, we are not worried that the corrected test influences the results in any way.*

Another point that can be improved is the selection of groups when analyzing parents' conditions. It could be done with easy clustering algorithms to check if their groups are correctly separated. Alternatively, a better explanation about why authors selected three groups (instead of four or two, for example) could be adequate.

*Thank you for pointing out that we should have explained this in a better way. We have now done that in both the description of the student background variables, and in the description of the results.*

*On page 7 we have added the following sentence:*

*“This division of parental education over three categories is also being used in the Netherlands Cohort Study on Education and leads to a division in categories that is not only relevant at the content level, but also provides us with large enough groups to have statistical power.”*

*Furthermore, on page 15 we have added the following sentence to the results:*

*“(Note that alternative specifications in which we use four categories of parental education (in a similar way as the Dutch Inspectorate of Education), or in which we use three categories which are not based on parental education, but on the indication (used for funding purposes) whether a child is a regular child, has a disadvantaged background or a very disadvantaged background, yield very similar results and the same conclusions.)”*

Typos: "the school closures and the COVID-1919-pandemic than others"
"paper comes down to 201819 students in 1178"
"This is implies that during the"

*Thank you, we have corrected the typos in the text and checked once more for other typos as well.*

Congratulations for your nice work.

*Thank you, and thank you once more for your valuable comments. We hope we have been able to deal with your comments in a satisfactory way.*
