# Supplementary material for: Sharp increase in inequality in education in times of the COVID-19-pandemic
Source: PLoS One. 2022 Feb 2;17(2):e0261114. doi: 10.1371/journal.pone.0261114 (PMC8809564; doi:10.1371/journal.pone.0261114)
Supplement: Attachment — Submitted filename: Response to reviewers_revision2.docx [file pone.0261114.s002.docx]

**Sharp increase in inequality in education in times of the COVID-19-pandemic**

**PLOS ONE**

November 2021

**Response to reviewer 2**

*First, let us express our gratitude for your positive words, and constructive and insightful comments and remarks. They have helped us a lot in revising and essentially improving the paper. We believe that we have been able to address all of your and their comments. Based on the comments we extended our discussion of the international literature on the role of the COVID-19 pandemic in children’s educational development, we performed additional robustness checks (e.g., school fixed effects) and clarified some of our tables and figures. We address the comments of the reviewer hereafter in more detail in a point-by-point response. We hope that you will be satisfied by the respective revisions and replies.*

This an interesting and very timely study on pertinent issues in education policy.

*Thank you.*

Below are some concerns I have about the study. Clarifying those would be helpful.

1. How does the sample used in the study compare to the overall school population in the Netherlands? In other words, how representative is the sample of Netherlands nationally? The authors refer to using a national sample in calculating weights. How do averages of various variables in the study sample compare to those in the national sample?

*Schools are only included in our sample if they gave permission to share their test scores with Statistics Netherlands. As a result, our sample is not an exact representation of the population of Dutch primary school students. We added Table 3 to the manuscript to show how our study sample compares to the full population. Overall, our sample contains an overrepresentation of one-parent households (19.00% versus 16.16% in the full population), students with a non-western migration background (20.82% versus 14.36%), students with low parental income (24.06% versus 21.20%), and an underrepresentation of students from which both parents work (68.47% versus 70.91%). Furthermore, schools in our sample tend to be larger schools located in more urbanized areas. As you mention already, we use inverse probability weights to minimize the impact of the selectivity of our sample.*

*Table 3. Representativeness of sample compared to full population on student and school background variables*

|  | *Full population* | *Sample* |
| --- | --- | --- |
| *Variables* | *Percentage* | *Percentage* |
| *Gender* |  |  |
| *Female* | *49.32* | *49.75* |
|  |  |  |
| *Migration background* |  |  |
| *Dutch & western migration background* | *82.22* | *75.95* |
| *Non-western migration background* | *17.51* | *24.04* |
| *Missing* | *0.27* | *0.01* |
|  |  |  |
| *Parental income* |  |  |
| *Low income* | *21.20* | *24.06* |
| *Medium income* | *53.38* | *50.54* |
| *High income* | *24.02* | *24.53* |
| *Missing* | *1.41* | *0.87* |
|  |  |  |
| *Parental education* |  |  |
| *Low educated* | *10.06* | *11.50* |
| *Medium educated* | *29.87* | *29.03* |
| *High educated* | *47.59* | *48.95* |
| *Missing* | *12.48* | *10.52* |
|  |  |  |
| *School size* |  |  |
| *Less than 141 students* | *36.71* | *28.95* |
| *Between 141 – 220 students* | *30.27* | *31.26* |
| *More than 220 students* | *33.01* | *39.79* |
|  |  |  |
| *School level pct of low educated parents* |  |  |
| *Below 5,5%* | *33.12* | *32.22* |
| *Between 5,5% and 12%* | *33.47* | *32.78* |
| *Above 12%* | *33.41* | *35.00* |
|  |  |  |
| *Urbanisation level* |  |  |
| *Low (< 500 adresses/km2)* | *7.86* | *5.63* |
| *Limited (500 – 1000 adresses/km2)* | *21.87* | *14.41* |
| *Medium (1000 – 1500 adresses/km2* | *17.58* | *12.50* |
| *Strong (1500 – 2500 adresses/km2)* | *30.93* | *32.66* |
| *Very strong (>=2500 adresses/km2)* | *21.77* | *34.81* |
|  |  |  |
| *Denomination* |  |  |
| *Public school* | *29.79* | *30.55* |
| *Schools based on philosophies* | *6.10* | *3.77* |
| *Schools based on religious beliefs* | *64.02* | *65.69* |
|  |  |  |
| *Observations* |  |  |
| *Total number* | *2,458,376* | *263,553* |
| *Note: N of students is based on the number of unique students in the years 2017/2018, 2018/2019 and 2019/2020* | | |

2. Relatedly, what proportion of total schools in Netherlands does the sample of schools, students and test records in Table 2 represent?

*Thank you for pointing out that this was not clear. In the schoolyear 2019/2020, we had a total number of 6174 primary schools in the Netherlands. The 1178 schools in our sample therefore comprise a proportion of 19% of the total number of schools.*

*Having said that, we realised that the distinction between test supplier and administrative system could be confusing (see your next question and our answer). Moreover, the distinction between the different administration systems is not relevant for our analyses. Therefore, we decided to no longer mention the names of the administration systems in our paper and to remove Table 2.*

3. On page 6 authors mention that “Test supplier CITO made a recalculation for all test scores to correct for this delayed testing.” Is CITO the only supplier that did this recalculation or did other mentioned suppliers did it too? What kind of impact can we expect this calculation to have on the scores, especially in comparison to other suppliers and to previous years scores? While authors cannot get to the actual calculation CITO did, some explanation would be helpful.

*Again: thank you for making us realise this was unclear. Based on your question we realised that there might be confusion between what a test supplier is and what an administrative system is. In the previous version of the paper, we made a distinction between test suppliers and administrative systems. All children in our sample made tests of a supplier called CITO. CITO is by far the largest test supplier in the Netherlands. The test scores of the CITO-tests are saved in an administration system. Schools can choose themselves which administration system they prefer. Our dataset is based on the data stored in three types of administration systems: CITO-LOVS, ParnasSys and ESIS. Former table 2 showed the number of schools per administration system.*

*However, based on your comment, we realised that the names CITO-LOVS (administration system) and CITO (test supplier) could confuse our readers. As explained in our answer to your previous question, we have now removed Table 2.*

*So as an answer to your questions 3: there is only* ***one*** *test supplier that we include in our analyses, namely CITO. Therefore, the scores of all students in our sample who performed the test after the summer were corrected. By correcting for the delay, the test supplier aimed to make the test scores comparable to the test scores of students who took the test before summer, to account for the extra time the students had until the test was taken. Note that the test supplier did not correct for the fact that this test was taken in the period of COVID-19 in any way, meaning that they did not correct for lower scores compared to previous years. To make sure our results are not biased by the differences in the timing of the test we also include a dummy in our regression analyses indicating whether a test was made before (=0) or after (=1) summer. Based on your comment, we improved our explanation of the differences in test scores before and after the summer on page 6.*

4. Do the authors have any information on teachers or school level variables? Does the study account for teacher and school level effects in their estimations? For example we can expect some teachers to do better in remote instruction that others. Are/Can the estimations account for this in any way?

*Thank you for this question. We do have information on school level variables, but unfortunately we do not have any information on the teachers. We agree that school and teacher level factors play a role in the extent to which the COVID-19 related school closings affected student learning. The association between student characteristics and learning gains might be driven by, for example, differences between schools serving different types of student populations. To account for this possibility, we have added a new analysis incorporating Fixed Effects at the school level. These can be found in Table 9 (split by grade level) on page 24 and Table 10 (pooled over all grades) on page 25. In this specification, we account for any (un)observed time invariant differences between schools that could have an effect on the strength of the relationship between student characteristics and the COVID-19 related reductions in learning gains. The results show that the estimations remain unaffected. The possibility that different types of teachers may differentially affect student learning gains unfortunately cannot be tested, as we do not have information on the teachers to whom students have been assigned.*

5. Parental Education: They mention on page 7 that “Parental education is defined as low
when the highest obtained degree of (one) the parents is in pre-vocati…..”. They mention further down in the text that they use the higher of the two parents educational attainment. Would it not be pertinent instead to also look at this my mother/father or by the parent with the larger share of child responsibilities? Perhaps, if we expect the parent with lower educational attainment to be more responsible for child caring, look at estimates along that margin?

*Thank you for this very relevant question. However, we believe that in our setup, parents’ highest obtained level of education is still the best proxy for children’s socioeconomic background for several reasons. One is that we cannot be certain as to which parent has the larger share of child responsibilities. Especially during the time of the pandemic and the increase in remote working, the balance between work and family life is likely to have shifted compared to previous years. This might render interpretation of the results difficult. Secondly, the administrative data on parents’ highest obtained level of education does not yet fully cover the entire population of the Netherlands. In combining the education data from both mother and father, the share of missing observations is reduced. Due to assortative mating there is a relatively high correlation between the highest obtained level of education of both mother and father. Hence, the education of one of the parents is also an indicator of the educational attainment of the other parent.*

*Nevertheless, you are right that it is instructive to look at the sensitivity of our results to running estimations including either the mother’s or father’s education, or both at the same time, instead of the highest level. The results of this exercise show that pooled over all grades, the main association between parental education and learning loss during the pandemic holds for all three subjects. For reading, maternal education enters more significantly than paternal education, while for the other subjects there is no difference. In the analyses by grade, we again see no differences for spelling and math. For reading, high paternal education appears to be more significantly related to learning losses in grades 3, 4, and 5. However, these results do not survive our standard robustness specifications. Despite the small differences in results based on paternal and maternal education and children’s reading, our overall conclusions do not change when paternal education or maternal education is used. Because of these results, as well as the aforementioned conceptual reasons, we strongly favour the models in which the highest level of parental education is used.*

6. The school closure periods mentioned on page 4 is not very long. How do authors see this in light of closure in other parts of the world e.g. U.S. where schools remained closed for extended periods of time? Can we expect more widening of gaps across different groups if closure remained longer?

*We agree with you that children in countries with longer school closings and less internet access might have experienced larger learning losses and larger inequalities because they experienced prolonged periods of limited and unequal excess to education. However, the current literature is inconclusive and more research is needed to draw pertinent conclusions on the association between the length of the lockdown and children’s educational progress. For example, a recent study in Italy by Contini et al (2021) reports larger learning losses (0.19 SD) than previous studies in the Netherlands (0.08 SD) by Engzell et al (2021). In Italy schools were closed for 15 weeks (one of the first and longest school closings in Europe). Moreover, Italy has one of the lowest share of households with a broadband connection and 12% of the students between 6 and 17 years old did not have access to a computer or digital tools at home in 2018/2019. However, contradicting the idea that longer school closings result in larger learning deficits, a study in Belgium (8.5 weeks of school closing) reports a reduction in mathematics scores of 0.19 SD which is similar in size to the effects found in Italy (15 weeks). Altogether, more research based on country comparisons is needed to be able to state that longer lockdowns result in larger learning deficits and an increase in educational inequalities. We have added a paragraph to reflect on country differences on page 4 and 5.*

7. During the time period of the closure, the vulnerable kids were apparently still allowed to attend schools. How does this, if at all, interact with the income and education of the households. Are authors able to identify students who continued attending during the closure?

*Thank you for this question. When the Dutch government decided to close schools, some children still went to school. The government prescribed that schools could allow ‘vulnerable’ children as well as children of parents with essential occupations at school. These rules on which children could and could not attend school were purposely vague and schools could mostly define their own policies. As a result, we do not know which children went to school and which children stayed at home. However, especially during the first lockdown, which is the subject of our study, schools were only open for emergencies and the number of children at school was very low. Based on a survey by the General Association of School Leaders about 5% of the students went to school during the first lockdown. Moreover, children who went to school during the school closings usually followed a program which was similar to the program of the children who stayed at home. We have now also added a few sentences on this on page 4. Finally, due to the various reasons to let children spend their day at school, the group of children at school was diverse and exceeded low socioeconomic status families. Since the number of children that attended school is not registered in our data, we cannot make a distinction between children who attended school and who did not attend school during the school closure so we cannot examine the differences in performances based on school attendance.*

*Whether the presence of a small group of children at school is problematic for the analyses depends on your goal. We argue that we estimate the effect of the Dutch educational policies during the pandemic on children’s education progress. Allowing a selective group of children at school is part of the educational policies and therefore not problematic for our analyses. Nevertheless, we acknowledge that school attendance by vulnerable groups might cause a bias if one wants to estimate the effect of distance education on educational progress. If a bias would occur, one might argue that the results reported in our studies underestimate the inequalities based on parents’ education and income since some inequalities might have been cushioned by school attendance. However, we argue that the bias would be small since the number of children allowed at school was small and the group of children relatively diverse. Moreover, the educational program of children at school and children at home was relatively similar.*

8. Results: Education literature generally finds that educational interventions bring a larger change in math and a smaller change in reading scores, partly because reading is not just dependent on what is taught in school but requires stronger input from home also. It would be nice to tie in that literature with the Math vs. Reading losses the authors estimates

*Thank you for the helpful literature suggestion. The idea that family environment plays an important role in the development of reading skills is supported by the finding that reading skills are less affected by the school closure. The relative importance of schools might be smaller for reading than for other subjects which explains the limited impact of the pandemic. In contrast, the limited increase in socioeconomic inequalities in reading skills is not in line with our expectations. Normally, family environment is an important source of inequality in reading skills and we would expect inequalities to rise when schools closed and the role of family environment increased. We have added a discussion on the differences between reading and mathematics before and during the pandemic in the Conclusions on page 32 and 33.*

9. How should we see migrants in terms of income and education? In other words what is the average education level of migrants in Netherlands and what income category should we expect them to fall into. In other words, more clarity on needed for the reader on analysis over migration vs. income or education.

*You are right that we should clarify to the international audience how students with a non-western migrant background should be seen in terms of parental education and household income. We have added the comparison of the share of low educated parents and low household income between students with a non-western migration background and native / western migrant students on page 8. The share of students with low educated parents and a low household income is higher for those with a non-western migration background than for those with a native / western migration background (26% vs. 6% for low parental education, 45% vs. 16% for low household income.*

10. It is not clear what Table 3 is showing. Is it the number of test score observations? If yes, why do we have decimals? If no, then are these some averages of test scores?

*In former Table 3 (Table 2 in the new revised version of the paper) we indeed show the number of observations (test records) per domain and per grade. We accidently used the European way to write this down. In continental Europe the meaning of punctuation marks is the exact opposite from Anglo-Saxon countries. In the Netherlands, and many other European countries, dots are used to separate large numbers while commas are used as decimal markers. We revised our punctuation across the manuscript to the Anglo-Saxon manners with commas to separate large numbers and dots as decimal markers. Hopefully this also clarifies the interpretation of Table 2.*

11. Figure 5, 6 and 7 need to be clearer. I could not understand what was being shown by each line.

*Thank you for pointing this out. We have first of all made these figures a bit bigger in the manuscript, such that they are better to read. Furthermore, as for explanation what information the figures show: Figures 5, 6 and 7 show the trends in learning gains per grade over time by plotting the unstandardized learning gains by cohort. Because our data does not go back equally far for all grades, the lines are of different length, which perhaps causes some confusion (see also table 1 on page 5). For grade 5, we have data for the grade 5 cohorts of 2017/2018 until 2019/2020, while for grade 2 we have data for students that were in grade 2 from the 2014/2015 academic year onwards. The main message from these figures is that we see a clear drop in learning gains for most grades during the 2019/2020 academic year for the three domains reading, spelling, and math. We have added some additional clarifying sentences regarding these figures on page 27.*

***Minor Comments***
1. Relevant study to cite on the effect of pandemic on student learning : <https://gpl.gsu.edu/download/student-achievement-growth-during-the-covid-19-pandemic-report-appendix/?wpdmdl=2101&refresh=614b7200638111632334336>

*Thank you for the useful suggestion. We included the report in our discussion of the literature on page 3.*

2. Contribution: I think in terms of contribution the authors need to think beyond the Dutch data. They emphasize on page 3 the comparison to other work that uses Dutch data. I would urge them to look at other studies, in different countries, that look at inequality in education outcomes during the pandemic and situate their study in the wider literature.

*We have improved the discussion of previous literature in the introduction (page 2 and 3). The new version of the introduction includes more references to the international literature and has been complemented with recent studies which appeared during the review process of our paper.*

3. The authors mention on page 3 their contribution compared to an existing study on Dutch data. I don’t think having an 18% vs 15% sample is a contribution, unless the new sample is more representative for some reason. The other points about having better and richer background info is certainly something to point out.

*In our enthusiasm to show the reader that we have a richer and better dataset to examine the consequences of the school closings on educational growth of primary school students in the Netherlands, we argued that our sample is also somewhat larger than the sample used by Engzell et al. Naturally, this is not the strongest argument. As the reviewer already states, our main improvement is based on the rich background information we have in our data. Based on this comment and the previous comment (minor 2) we have rewritten the paragraph on page 3. We now situate our study in the international literature. We emphasize now how the Dutch context offers, due to standardized testing, unique opportunities to study the consequences of the school closings. Moreover, we emphasize that we have, compared to previous studies in the Netherlands, better information on students’ background characteristics.*

Typos
*Thank you for pointing them out. We have now corrected the typos.*

*In addition to your comments, we have also substantially shortened the results section that contained a lot of repetition, and have removed some more typographical errors from the paper.*

*Thank you once more for your valuable comments. We hope we have been able to deal with your comments in a satisfactory way.*
